# Methylmalonic Acid and Homocysteine as Indicators of Vitamin B12 Deficiency in Patients with Gastric Cancer after Gastrectomy

**DOI:** 10.3390/nu11020450

**Published:** 2019-02-21

**Authors:** Sae-Mi Lee, Jongwon Oh, Mi-Ryung Chun, Soo-Youn Lee

**Affiliations:** 1 GC genome, 107, Ihyeon-ro 30beon-gil, Giheung-gu, Yongin-si, Gyeonggi-do 16924, Korea; saemi.lee@greencross.com; 2 Department of Laboratory Medicine and Genetics, Samsung Medical Center, Sungkyunkwan University School of Medicine, 81 Irwon-ro, Gangnam-gu, Seoul 06351, Korea; jongwon1234.oh@samsung.com (J.O.); miryung.chun@samsung.com (M.-R.C.)

**Keywords:** methylmalonic acid, homocysteine, vitamin B12, gastric cancer, gastrectomy

## Abstract

Vitamin B12 deficiency is a common complication in patients after gastrectomy. Elevated methylmalonic acid (MMA) and homocysteine are better indications of vitamin B12 deficiency than vitamin B12 serum level. We compared MMA and homocysteine levels of patients with gastric cancer after gastrectomy (*n* = 151) with controls (*n* = 142) and evaluated the prevalence of vitamin B12 deficiency using MMA and homocysteine in patients. MMA and homocysteine levels were significantly higher (*p* < 0.05) in patients with gastric cancer after gastrectomy. Of the 151 patients assessed after gastrectomy, 32 patients (21.2%) were vitamin B12 deficient as defined by serum MMA levels > 350 nmol/L, and 8 patients (5.3%) were vitamin B12 deficient as defined by serum homocysteine levels > 15 μmol/L. Both MMA and homocysteine levels were elevated in 7 patients. Among 33 patients with elevated MMA or homocysteine levels, 8 patients (24.2%) were vitamin B12 deficient based on a serum vitamin B12 level < 200 pg/mL. Additionally, levels of MMA and homocysteine were compared pre- and post-gastrectomy in 27 patients. The median MMA level was higher in patients with post-gastrectomy compared to pre-gastrectomy, while the median serum homocysteine level was not significantly different. These results indicate that using serum vitamin B12 levels alone may fail to detect vitamin B12 deficiency. Additional assessments of MMA and homocysteine levels are useful to evaluate possible vitamin B12 deficiency in patients who underwent a gastrectomy, and MMA is a better indicator than homocysteine to detect early changes in vitamin B12 levels.

## 1. Introduction

Vitamin B12 is essential for DNA synthesis and maintenance of normal hematologic and neurologic functions [1]. It acts as a cofactor for two enzymatic reactions: the conversion of methylmalonic acid (MMA) to succinic acid and the synthesis of methionine from homocysteine. Acquired vitamin B12 deficiency or congenital vitamin B12 metabolism disorders impair both enzymatic reactions. Acquired vitamin B12 deficiencies are caused by inadequate dietary intake, malabsorption, or other medical conditions such as pernicious anemia [2]. A gastrectomy is a well-known cause of vitamin B12 deficiency [3]. A gastrectomy results in lack of intrinsic factors and causes gradual depletion of vitamin B12 stores, with deficiency occurring 1–2 years post-surgery [3,4]. Gastric cancer is a common malignancy in Korea [5], and patient survival has increased as a result of early detection and improved treatment protocols. Therefore, screening and treatment of vitamin B12 deficiency have become more important for improving the quality of life in gastric cancer patients [6]. The serum vitamin B12 assay currently used as the initial routine diagnostic test has several limitations including poor agreement between laboratories, methods, or platforms and low sensitivity and specificity [4,7]. To overcome these limitations, several markers associated with vitamin B12 metabolism could be used in place of a single vitamin B12 assay. MMA and homocysteine, which accumulate in the absence of adequate vitamin B12, are sensitive and specific markers of vitamin B12 function and are used to diagnose vitamin B12 deficiency at an early stage and monitor treatment effects [8]. Vitamin B12 deficiency may be underestimated in patients with gastric cancer who underwent gastrectomy when assessed using measurements of vitamin B12 levels because serum vitamin B12 levels do not reflect functional vitamin B12 deficiency.

MMA and homocysteine levels have been evaluated as early markers of vitamin B12 deficiency in different groups including the general population, elderly, infants, pregnant women, and healthy women [7,9,10,11,12,13]. However, studies measuring both MMA and homocysteine in patients who underwent gastrectomy are limited. Therefore, in this study, we compared MMA and homocysteine levels in patients after gastrectomy with healthy controls and determined the prevalence of vitamin B12 deficiency using MMA and homocysteine levels in patients after gastrectomy. We also compared MMA and homocysteine levels in patients before and after gastrectomy.

## 2. Materials and Methods

### 2.1. Study Population

This study enrolled 151 Korean adult patients with gastric cancer who underwent gastrectomy and were followed up at Samsung Medical Center (Seoul, Korea), a tertiary care hospital. Individuals for the control group were selected from adults without any clinical symptoms or signs of illness that had visited the health promotion center for a medical checkup. Inclusion criteria for the control group were as follows: (1) No medical history of diseases such as malignancy, renal and thyroid dysfunction, psoriasis, and diabetes, and (2) no history of certain medications that have been associated with elevated homocysteine levels (anticonvulsant agents, folate antagonists, cholestyramine, metformin, thiazide diuretics, cyclosporine, levodopa). Demographic data including age, sex, operation date, and medications prescribed were obtained from electronic medical records. Hematologic and biochemical parameters including hemoglobin (Hb), mean corpuscular volume (MCV), creatinine, serum total protein, albumin, aspartate aminotransferase (AST), and alanine aminotransferase (ALT) were measured concurrently with MMA and homocysteine. The following levels were used to define vitamin B12 deficiency based on previously published research: (1) MMA level > 350 nmol/L [14], (2) homocysteine level > 15 μmol/L [15], and (3) serum vitamin B12 level < 200 pg/mL. Among patients who underwent gastrectomy, vitamin B12 was also measured in patients whose MMA or homocysteine levels were above the defined deficiency level. In addition, MMA and homocysteine were measured before and after gastrectomy in 27 patients who survived for more than five years after gastrectomy. Among them, 6 patients underwent total gastrectomy and 21 patients underwent subtotal gastrectomy. Of 6 patients who underwent total gastrectomy, 5 were treated with an intramuscular injection of cobamamide (1000 μg once a month to once a year, varied by patients) and 1 was treated with oral administration of cyanocobalamin after gastrectomy (0.25 mg twice a day). While, 27 patients who underwent subtotal gastrectomy were not treated. This study was approved by the Institutional Review Board of Samsung Medical Center (IRB No. SMC 2015-07-027-006) and written consents were obtained for subjects who need additional blood collection.

### 2.2. Analytical Procedures

Blood samples were collected in red stoppered plain tubes (Becton Dickinson Co., Franklin Lakes, NJ, USA). Serum was separated from whole blood and immediately stored at −20 °C until assayed. Serum MMA and homocysteine levels were measured on an Acquity ultra-performance liquid chromatography (UPLC) (Waters Corporation, Milford, MA, USA) connected to a Xevo TQ-S tandem mass spectrometer (Waters Corporation, Milford, MA, USA) using the modified method described by Hempen et al. [16]. Intra- and inter-assay imprecision were both <10% of the coefficient of variation. The accuracy of the homocysteine assay was ensured using the Proficiency Testing/Quality Management program of the United States College of American Pathologists survey. The assay detection ranges were 45.0–2810.0 nmol/L for MMA and 5.0–60.0 μmol/L for homocysteine.

Serum vitamin B12 levels were measured with a chemiluminescent immunoassay using an ADIVA Centaur XP (Siemens Diagnostics, NY, USA). Hb and MCV were analyzed using Sysmex XE-2100 and XN-9000 (Sysmex, Kobe, Japan) hematology analyzers, and creatinine, serum total protein, albumin, AST, and ALT levels were measured using a Roche modular analyzer (Roche Diagnostics Corp., Indianapolis, IN, USA) according to the manufacturer’s instructions.

### 2.3. Statistical Analysis

Data were analyzed using SPSS software v23.0 (SPSS Inc. 233 S. Chicago, IL, USA). The independent sample t-test and Wilcoxon rank sum test were used to assess the significant differences between gastrectomy patients and controls for continuous values. Categorical variables were compared using the Chi-square test. To assess differences in MMA and homocysteine levels measured before and after gastrectomy, the Wilcoxon signed rank test was used. *p*-values less than 0.05 were considered statistically significant.

## 3. Results

This study included 151 patients that had a previous gastrectomy (87 male and 64 female) and 142 healthy controls (87 male and 55 female). The median follow-up period was 35 months with a range of 1–166 months. The demographic characteristics, hematologic, and biochemical results of patients and controls are shown in Table 1. Age, Hb, MCV, albumin, and total cholesterol were significantly higher in the control group. There were no significant differences between gastrectomy patient and healthy control groups in sex, creatinine, total protein, AST, and ALT.

**Figure 1:**
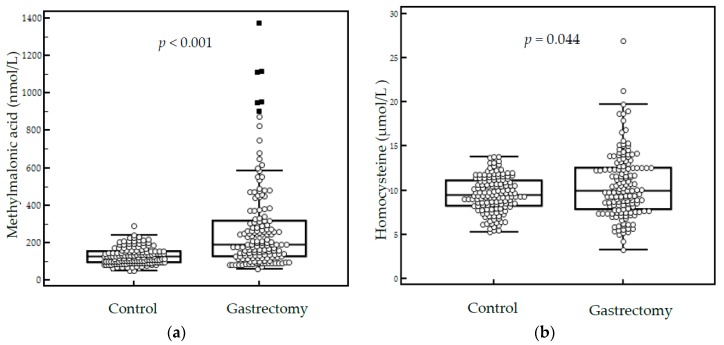
Serum methylmalonic acid (**a**) and homocysteine levels (**b**) in the control group (*n* = 142) and gastrectomy patient group (*n* = 151).

The median serum MMA level was significantly higher in gastrectomy patients than in controls (191.3 vs. 125.7 nmol/L, *p* < 0.001), and the median serum homocysteine level was also significantly higher in gastrectomy patients (10.0 vs. 9.5 μmol/L, *p* = 0.044) (Figure 1). Additionally, we compared 109 gastrectomy patients and 134 controls below 65 years old. There were no significant differences in age (53 vs. 52, *p* = 0.891). The median MMA level was higher for patients after gastrectomy (188.3 vs. 124.3 nmol/L, *p* < 0.001), but the median serum homocysteine level was not significantly different (9.4 vs. 9.5, *p* = 0.804).

Of the 151 gastrectomy patients, 32 patients (21.2%) were vitamin B12 deficient based on serum MMA levels >350 nmol/L, and eight patients (5.3%) were vitamin B12 deficient based on serum homocysteine levels > 15 μmol/L. MMA and homocysteine were both elevated in seven patients. Among the 33 patients with elevated MMA or homocysteine levels, eight patients (24.2%) had low serum vitamin B12 levels. Therefore, using MMA and homocysteine as indicators of vitamin B12 deficiency detects more vitamin B12-deficient patients. No individuals in the healthy control group had elevated MMA or homocysteine levels.

**Figure 2:**
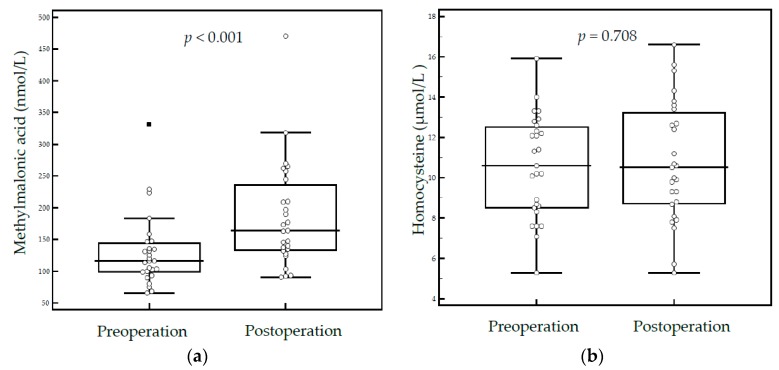
Serum methylmalonic acid (**a**) and homocysteine levels (**b**) in patients before and after gastrectomy (*n* = 27).

In 27 patients who survived for more than five years after gastrectomy, the median MMA level was higher in patients after gastrectomy compared to before a gastrectomy (163.8 vs. 116.2 nmol/L, *p* < 0.001). However, serum homocysteine levels were not significantly different after gastrectomy (10.5 vs. 10.6, *p* = 0.708) (Figure 2). Although no individuals had elevated MMA after gastrectomy, an increase was observed comparing MMA levels pre- and post-gastrectomy.

## 4. Discussion

Early detection of vitamin B12 deficiency is clinically important because the neurologic symptoms associated with vitamin B12 deficiency cannot be reversed and pernicious anemia will occur if diagnosed late but can be prevented with a vitamin B12 supplement [1,17]. There is no gold standard for diagnosing vitamin B12 deficiency. Due to limitations of assays that directly measure vitamin B12, metabolites that accumulate as a result of vitamin B12 deficiency are now measured in clinical settings [18].

In this study, we determined the prevalence of vitamin B12 deficiency using MMA and homocysteine levels. The prevalence of vitamin B12 deficiency was 21.9% (33/151) of gastrectomy patients as defined by elevated MMA or homocysteine levels. Of those patients, only 24.2% (8/33) were vitamin B12 deficient based on serum vitamin B12 levels < 200 pg/mL. Therefore, by measuring MMA and homocysteine levels, we found vitamin B12 deficiencies that were not detected by measuring vitamin B12 alone. These findings suggest that only measuring vitamin B12 could miss vitamin B12 deficiency in some patients.

We also evaluated the utility of MMA and homocysteine as follow-up markers after gastrectomy by comparing MMA and homocysteine levels in patients before and after gastrectomy. Although a small number of patients were included, there was a significant increase in MMA levels after gastrectomy. However, homocysteine was not significantly increased post-gastrectomy compared to pre-gastrectomy. This suggests that MMA could be a better follow-up marker than homocysteine in patients after gastrectomy. The superiority of MMA sensitivity to that of homocysteine is well known. An elevated MMA level is more sensitive and specific for vitamin B12 deficiency than an elevated homocysteine level because homocysteine is also increased in folate deficiency, vitamin B6 deficiency and other conditions such as renal failure and hypothyroidism [19,20].

It was reported that a vitamin B12 deficiency prevalence was 55.6% (40/72 patients) at a median follow-up of 29 months after gastrectomy based on vitamin B12 levels < 200 pg/mL [21]. In this study, homocysteine was significantly elevated in patients after gastrectomy compared to healthy controls (15.1 vs. 12.5 μmol/L, *p* = 0.016). Another study reported a vitamin B12 deficiency prevalence of 28.5% (184/645 patients) at a median follow-up of 24 months in patients after gastrectomy based on vitamin B12 levels < 200 pg/mL [22]. They report that homocysteine was significantly elevated in the vitamin B12 deficient group compared to the vitamin B12 non-deficient group (12.9 vs. 12.0 μmol/L, *p* = 0.006). Sakuta et al. reported homocysteine was higher in gastrectomy patients than in controls (11.7 vs. 9.3 μmol/L, *p* = 0.011) [23]. In our study, the prevalence of vitamin B12 deficiency was 21.9% (33/151 patients) based on elevated MMA or homocysteine levels at a median follow-up of 35 months after gastrectomy. MMA and homocysteine were significantly elevated in gastrectomy patients compared to healthy controls (MMA: 191.3 vs. 125.7 nmol/L, *p* < 0.001; homocysteine: 10.0 vs. 9.5 μmol/L, *p* = 0.044). These studies reported that homocysteine was elevated in gastrectomy patients compared to control groups, which is in accordance with the results of this study. But we could not rule out the effect of older age in gastrectomy patients group. Subgroup analysis in 109 gastrectomy patients and 134 controls below 65 years old, homocysteine level was not significantly different. This result may support the low sensitivity of homocysteine.

The prevalence of vitamin B12 deficiency varies by study because of differences in the definition of vitamin B12 deficiency and different cut-off levels, operation types, and follow-up periods. A worldwide consensus on the definition of vitamin B12 deficiency is needed to accurately evaluate the prevalence of vitamin B12 deficiency in gastrectomy patients. Using different cut-off levels to define vitamin B12 deficiency could affect the prevalence of vitamin B12 deficiency. For instance, it was reported that an MMA cut-off level of 0.376 nmol/L resulted in a prevalence of 2.4%, whereas with a cut-off level of 0.271 nmol/L the prevalence was 5.9% in the general population [18]. Further studies to define standardized cut-off levels are needed. Additionally, patient composition depending on the extent of the gastrectomy could affect the prevalence of vitamin B12 deficiency. As such, in previous studies, most vitamin B12 deficiencies occurred in patients who had undergone total gastrectomy. Lim et al. reported that at 48 months after gastrectomy, the incidence of vitamin B12 deficiency was 3.2% in patients that had a Billroth I subtotal gastrectomy, 7.5% in patients that had a Billroth II subtotal gastrectomy, and 76.9% in patients that had total gastrectomy [24]. This study also reported that the incidences of vitamin B12 deficiency in patients who underwent total gastrectomy were 0%, 16.1%, 50.0%, and 76.9% at 12, 24, 36, and 48 months after gastrectomy, respectively. Therefore, the incidence of vitamin B12 deficiency increased as time increased after gastrectomy.

Our study had several limitations. First, it was difficult to evaluate the accurate prevalence of vitamin B12 deficiency in patients with a gastrectomy due to the absence of a gold standard and various sensitivity and specificity of each diagnostic marker. High concentration of MMA or homocysteine can serve only a "possible” diagnosis of vitamin B12 deficiency. Therefore, vitamin B12 deficiency defined by MMA and homocysteine levels could detect patients with the potential for vitamin B12 deficiency. Second, it was a retrospective study and some information was unavailable. For instance, information on the use of dietary supplements, vitamins, or medications from another hospital was not collected. Third, we did not classify patients based on the extent of resection or history of chemotherapy, and the MMA and homocysteine measurements were done at various times after gastrectomy. Fourth, serum vitamin B12 levels were not measured in all patients and controls due to lack of sample volume. However, this study provides valuable information to the field because it is the first to simultaneously measure MMA and homocysteine levels in patients after gastrectomy and also the first to evaluate MMA and homocysteine as early markers of vitamin B12 deficiency in gastrectomy patients.

## 5. Conclusions

In conclusion, we simultaneously measured serum MMA and homocysteine levels and evaluated the prevalence of vitamin B12 deficiency in patients who underwent gastrectomy vs. controls. The measurement of MMA and homocysteine enabled detection of patients with possible vitamin B12 deficiency that may be missed by using the measurement of vitamin B12 alone. Further studies using various markers associated with vitamin B12 metabolism are needed to define the accurate prevalence of vitamin B12 deficiency and comprehensively and prospectively assess the utility of MMA and homocysteine as early markers of vitamin B12 deficiency in patients who underwent gastrectomy.

## Figures and Tables

**Table 1:** Comparison of demographic characteristics and biochemical markers between the control group (*n* = 142) and the gastrectomy patient group (*n* = 151).

Parameter	Controls (*n* = 142)	Gastrectomy Patients (*n* = 151)	*p*-Value
Age (years)	52 (33–75)	56 (32–78)	0.001 ^a^
Sex			0.525 ^b^
Male (n)	87	87	
Female (n)	55	64	
Hemoglobin (g/dL)	14.5 (1.3)	12.4 (1.9)	<0.001 ^c^
MCV (fL)	92.5 (85.3–102.9)	90.3 (63.3–111.0)	<0.001 ^a^
Creatinine (mg/dL)	0.84 (0.55–0.97)	0.84 (0.55–1.42)	0.159 ^a^
Total protein (g/dL)	7.0 (6.3–7.9)	7.0 (3.8–8.2)	0.568 ^a^
Albumin (g/dL)	4.6 (4.1–5.1)	4.3 (2.5–5.0)	<0.001 ^a^
Total cholesterol (mg/dL)	195.9 (32.8)	168.3 (33.1)	<0.001 ^c^
AST (U/L)	23 (12–81)	24 (13–123)	0.099 ^a^
ALT (U/L)	21 (7–97)	20 (7–93)	0.115 ^a^
MMA (nmol/L)	125.7 (48.4–291.5)	191.3 (60.2–1374.6)	<0.001 ^a^
Homocysteine (μmol/L)	9.5 (5.3–13.8)	10.0 (3.3–26.9)	0.044 ^a^

Abbreviations: ALT, alanine aminotransferase; AST, aspartate aminotransferase; MCV, mean corpuscular volume; MMA, methylmalonic acid. ^a^ results are presented as median (range) with *p*-values from a Wilcoxon rank sum test; ^b^ *p*-values from the chi-square test; ^c^ results are presented as mean (standard deviation) with *p*-values from student t-test.
